# 4,6-Dihy­droxy-4,6-dimethyl-1,3-diazinane-2-thione

**DOI:** 10.1107/S160053681103145X

**Published:** 2011-08-11

**Authors:** Khatira N. Aliyeva, Abel M. Maharramov, Mirze A. Allahverdiyev, Atash V. Gurbanov, Iván Brito

**Affiliations:** a Department of Organic Chemistry, Baku State University, Baku, Azerbaijan; b Departamento de Química, Facultad de Ciencias Básicas, Universidad de Antofagasta, Casilla 170, Antofagasta, Chile

## Abstract

In the title compound, C_6_H_12_N_2_O_2_S, the heterocyclic ring has a sofa conformation. The mol­ecular conformation is stabilized by an intra­molecular O—H⋯O hydrogen-bond inter­action with graph-set motif *S*(6). In the crystal, mol­ecules are linked by O—H⋯S, N—H⋯S and N—H⋯O hydrogen-bond inter­actions, forming an extended two-dimensional framework parallel to the *ac* plane.

## Related literature

For the preparation of pyrimidines by reactions of 1,3-dicarbonyl compounds (*e.g.* ethyl acetoacetate, acetyl­acetone) with urea, thio­urea, guanidine, see: Barton & Ollis (1979 ▶). For hydrogen-bond motifs, see: Bernstein *et al.* (1995 ▶). For ring conformations, see: Cremer & Pople (1975 ▶).
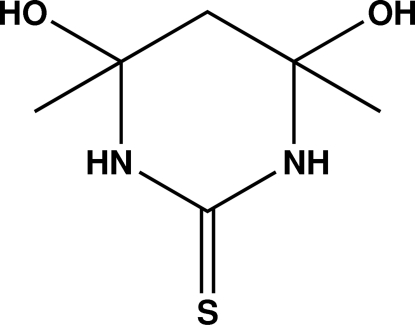


## Experimental

### 

#### Crystal data

C_6_H_12_N_2_O_2_S
*M* *_r_* = 176.24
Triclinic, 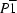

*a* = 5.2425 (4) Å
*b* = 8.7047 (6) Å
*c* = 9.4370 (7) Å
α = 74.812 (1)°
β = 88.670 (1)°
γ = 79.708 (1)°
*V* = 408.80 (5) Å^3^
*Z* = 2
Mo *K*α radiation
μ = 0.35 mm^−1^
*T* = 296 K
0.30 × 0.20 × 0.20 mm

#### Data collection

Bruker APEXII CCD diffractometer
Absorption correction: multi-scan (*SADABS*; Sheldrick, 2003 ▶) *T*_min_ = 0.903, *T*_max_ = 0.934
4260 measured reflections
1760 independent reflections
1557 reflections with *I* > 2σ(*I*)
*R*_int_ = 0.012

#### Refinement

*R*[*F* ^2^ > 2σ(*F* ^2^)] = 0.029
*wR*(*F* ^2^) = 0.080
*S* = 1.00
1760 reflections
102 parameters
H-atom parameters constrained
Δρ_max_ = 0.36 e Å^−3^
Δρ_min_ = −0.17 e Å^−3^

### 

Data collection: *APEX2* (Bruker, 2005 ▶); cell refinement: *SAINT-Plus* (Bruker, 2001 ▶); data reduction: *SAINT-Plus*; program(s) used to solve structure: *SHELXTL* (Sheldrick, 2008 ▶); program(s) used to refine structure: *SHELXTL*; molecular graphics: *OLEX2* (Dolomanov *et al.*, 2009 ▶); software used to prepare material for publication: *SHELXTL*.

## Supplementary Material

Crystal structure: contains datablock(s) I, global. DOI: 10.1107/S160053681103145X/bt5602sup1.cif

Structure factors: contains datablock(s) I. DOI: 10.1107/S160053681103145X/bt5602Isup2.hkl

Supplementary material file. DOI: 10.1107/S160053681103145X/bt5602Isup3.cml

Additional supplementary materials:  crystallographic information; 3D view; checkCIF report

## Figures and Tables

**Table 1:** Hydrogen-bond geometry (Å, °)

*D*—H⋯*A*	*D*—H	H⋯*A*	*D*⋯*A*	*D*—H⋯*A*
O1—H1*O*⋯O2	0.88	1.98	2.727 (2)	143
O2—H2*O*⋯S1^i^	0.88	2.37	3.249 (1)	173
N1—H1*N*⋯S1^ii^	0.92	2.60	3.414 (1)	149
N2—H2*N*⋯O1^iii^	0.92	2.18	3.074 (2)	164

Symmetry codes: (i) 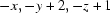; (ii) 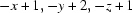; (iii) 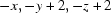.

**Fig. 1.**
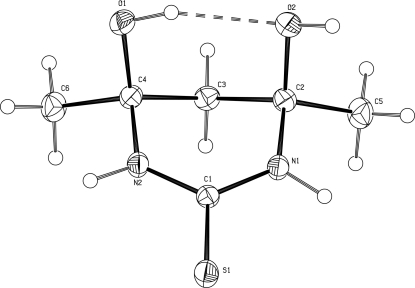
The structure of (I) showing the atom numbering scheme. The hydrogen bond is showing as dotted line. Displacement ellipsoids are drawn at 30% probability level.

**Fig. 2.**
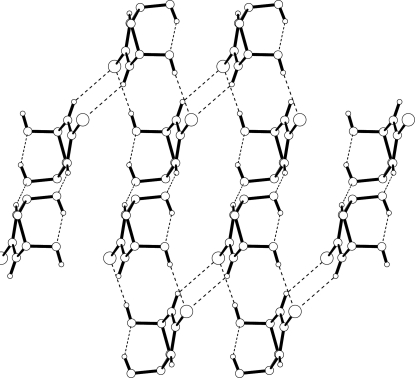
Part of the crystal structure showing O—H···S; N—H···S & N—H···O hydrogen-bond interactions parallel to ab plane. The methyl groups and the H atoms on C3 atom have been omitted for clarity.

## supplementary crystallographic information

### Comment

The biological activity of pyrimidine derivatives attracts great interest to their synthesis. Their derivatives play important part in the functions of the human body. Pyrimidine structural fragment is included into quite a number of natural substances (nucleic acids, vitamin B1), into synthetic medicinals (barbiturates), into chemotherapeutic preparations (fluorouracil). In preparation of pyrimidines are widely used reactions of 1,3-dicarbonyl compounds (*e.g.* ethyl acetoacetate, acetylacetone) with urea, thiourea, guanidine *etc* (Barton & Ollis, 1979). In the title compound (I), C_6_H_12_N_2_O_2_S, the heterocyclo ring has a sofa conformation, (Q_T_= 0.459 (13) Å, θ= 127.52 (7)°, φ_2_ = 59.54 (4)°, (Cremer & Pople, 1975). The molecular conformation is stabilized by one intramolecular O—H···O hydrogen-bond interaction with set graph motif S(6) (Bernstein, *et al.* 1995). In the crystal the molecules are linked by O—H···S, N—H···S, N—H···O hydrogen-bond interactions forming an extended two-dimensional framework parallel to *ab* plane, Table 1, Fig. 2.

### Experimental

On the anhydrous ethanol (40 ml) added 18 gram (0.783 mol) small pieces of metallic sodium and wasvigorously stirred until sodium fully reacted with ethanol. Then on theobtained solution was added 10 gram (0.1 mol) of acetylacetone and 7.4 gram (0.1 mol) ofthiourea. Reaction mixture was stirred two hour in room temperature. Then 120 ml distilled water added on reaction mixture and neutralized with 5 ml ofglacial acetic acid. Precipitated unreacted part of thiourea was filtered ofand the obtained filtrate stayed in -10 °C. After two days obtained single crystals of 4,6-dihydroxy-4,6-dimethyltetrahydropyrimidine-2(1*h*)-thione was collected. Yield 6 gram (42%), m.p. 254–255 °C.

^1^H NMR(300 MHz, DMSO-d6) δ 1.32 (s, 6H, 2CH_3_), 1.71–2.05 (m, 2H,CH_2_), 3.52 (s, 2H, 2OH), 6.16 (s, 1H, NH), 8.67 (s, 1H, NH). ^13^CNMR (75 MHz, DMSO-d6) δ 28.40,43.63, 78.98, 79.07, 175.23, 175.31

### Refinement

All H-atoms were placed in calculated positions [C—H = 0.96 to 0.97 Å, *U*_iso_(H) =1.2 to 1.5 *U*_eq_(C), O—H = 0.88 Å, *U*_iso_(H) =1.5 *U*_eq_(O) and N—H = 0.92 Å, *U*_iso_(H)=1.2 *U*_eq_(N)] and were included in the refinement in the riding model approximation.

### Crystal data

**Table:**

C_6_H_12_N_2_O_2_S	*Z* = 2
*M**_r_* = 176.24	*F*(000) = 188
Triclinic, *P*1	*D*_x_ = 1.432 Mg m^−^^3^
Hall symbol: -P 1	Mo *K*α radiation, λ = 0.71073 Å
*a* = 5.2425 (4) Å	Cell parameters from 2799 reflections
*b* = 8.7047 (6) Å	θ = 2.2–28.4°
*c* = 9.4370 (7) Å	µ = 0.35 mm^−^^1^
α = 74.812 (1)°	*T* = 296 K
β = 88.670 (1)°	Needle, colourless
γ = 79.708 (1)°	0.30 × 0.20 × 0.20 mm
*V* = 408.80 (5) Å^3^	

### Data collection

**Table:**

Bruker APEXII CCD diffractometer	1760 independent reflections
Radiation source: fine-focus sealed tube	1557 reflections with *I* > 2σ(*I*)
graphite	*R*_int_ = 0.012
φ and ω scans	θ_max_ = 27.0°, θ_min_ = 2.2°
Absorption correction: multi-scan (*SADABS*; Sheldrick, 2003)	*h* = −6→6
*T*_min_ = 0.903, *T*_max_ = 0.934	*k* = −11→11
4260 measured reflections	*l* = −12→12

### Refinement

**Table:**

Refinement on *F*^2^	Primary atom site location: structure-invariant direct methods
Least-squares matrix: full	Secondary atom site location: difference Fourier map
*R*[*F*^2^ > 2σ(*F*^2^)] = 0.029	Hydrogen site location: difference Fourier map
*wR*(*F*^2^) = 0.080	H-atom parameters constrained
*S* = 1.00	*w* = 1/[σ^2^(*F*_o_^2^) + (0.0543*P*)^2^ + 0.0583*P*] where *P* = (*F*_o_^2^ + 2*F*_c_^2^)/3
1760 reflections	(Δ/σ)_max_ = 0.001
102 parameters	Δρ_max_ = 0.36 e Å^−^^3^
0 restraints	Δρ_min_ = −0.17 e Å^−^^3^

### Special details

**Table:**

Geometry. All e.s.d.'s (except the e.s.d. in the dihedral angle between two l.s. planes) are estimated using the full covariance matrix. The cell e.s.d.'s are taken into account individually in the estimation of e.s.d.'s in distances, angles and torsion angles; correlations between e.s.d.'s in cell parameters are only used when they are defined by crystal symmetry. An approximate (isotropic) treatment of cell e.s.d.'s is used for estimating e.s.d.'s involving l.s. planes.
Refinement. Refinement of *F*^2^ against ALL reflections. The weighted *R*-factor *wR* and goodness of fit *S* are based on *F*^2^, conventional *R*-factors *R* are based on *F*, with *F* set to zero for negative *F*^2^. The threshold expression of *F*^2^ > σ(*F*^2^) is used only for calculating *R*-factors(gt) *etc*. and is not relevant to the choice of reflections for refinement. *R*-factors based on *F*^2^ are statistically about twice as large as those based on *F*, and *R*- factors based on ALL data will be even larger.

### Fractional atomic coordinates and isotropic or equivalent isotropic displacement parameters (Å^2^)

**Table:**

	*x*	*y*	*z*	*U*_iso_*/*U*_eq_	
O1	−0.13693 (17)	0.81379 (12)	0.96252 (10)	0.0356 (2)	
H1O	−0.2022	0.8234	0.8749	0.053*	
O2	−0.14367 (17)	0.74017 (12)	0.69909 (10)	0.0366 (2)	
H2O	−0.1954	0.7791	0.6065	0.055*	
N1	0.2210 (2)	0.86785 (12)	0.64127 (11)	0.0280 (2)	
H1N	0.2746	0.8901	0.5457	0.034*	
N2	0.2262 (2)	0.92771 (12)	0.86480 (11)	0.0279 (2)	
H2N	0.2349	1.0033	0.9160	0.033*	
S1	0.35950 (7)	1.14664 (4)	0.63906 (3)	0.03452 (13)	
C1	0.2615 (2)	0.96882 (14)	0.72002 (13)	0.0244 (2)	
C2	0.1320 (2)	0.71395 (14)	0.70201 (13)	0.0273 (3)	
C3	0.2177 (2)	0.65313 (14)	0.86236 (13)	0.0285 (3)	
H3A	0.4050	0.6213	0.8688	0.034*	
H3B	0.1433	0.5581	0.9075	0.034*	
C4	0.1366 (2)	0.78007 (15)	0.94675 (13)	0.0269 (3)	
C5	0.2428 (3)	0.59777 (17)	0.61166 (16)	0.0391 (3)	
H5A	0.1869	0.6437	0.5109	0.059*	
H5B	0.1827	0.4971	0.6486	0.059*	
H5C	0.4287	0.5790	0.6183	0.059*	
C6	0.2540 (3)	0.72820 (18)	1.10103 (14)	0.0365 (3)	
H6A	0.1999	0.8124	1.1494	0.055*	
H6B	0.4397	0.7081	1.0962	0.055*	
H6C	0.1966	0.6312	1.1551	0.055*	

### Atomic displacement parameters (Å^2^)

**Table:**

	*U*^11^	*U*^22^	*U*^33^	*U*^12^	*U*^13^	*U*^23^
O1	0.0260 (4)	0.0510 (6)	0.0316 (5)	−0.0065 (4)	0.0043 (4)	−0.0147 (4)
O2	0.0294 (5)	0.0502 (6)	0.0331 (5)	−0.0117 (4)	−0.0005 (4)	−0.0128 (4)
N1	0.0366 (5)	0.0270 (5)	0.0231 (5)	−0.0107 (4)	0.0039 (4)	−0.0081 (4)
N2	0.0347 (5)	0.0276 (5)	0.0236 (5)	−0.0084 (4)	0.0025 (4)	−0.0091 (4)
S1	0.0502 (2)	0.02887 (19)	0.02849 (18)	−0.01687 (14)	0.00523 (14)	−0.00838 (13)
C1	0.0229 (5)	0.0250 (6)	0.0256 (6)	−0.0036 (4)	0.0006 (4)	−0.0076 (4)
C2	0.0294 (6)	0.0256 (6)	0.0296 (6)	−0.0077 (5)	0.0026 (5)	−0.0104 (5)
C3	0.0303 (6)	0.0249 (6)	0.0293 (6)	−0.0059 (5)	0.0021 (5)	−0.0048 (5)
C4	0.0252 (6)	0.0309 (6)	0.0242 (6)	−0.0048 (4)	0.0018 (4)	−0.0067 (5)
C5	0.0509 (8)	0.0324 (7)	0.0400 (7)	−0.0099 (6)	0.0072 (6)	−0.0188 (6)
C6	0.0388 (7)	0.0420 (7)	0.0256 (6)	−0.0044 (6)	−0.0037 (5)	−0.0050 (5)

### Geometric parameters (Å, °)

**Table:**

O1—C4	1.4237 (14)	C2—C5	1.5185 (17)
O1—H1O	0.8800	C3—C4	1.5211 (17)
O2—C2	1.4223 (15)	C3—H3A	0.9700
O2—H2O	0.8800	C3—H3B	0.9700
N1—C1	1.3365 (15)	C4—C6	1.5166 (17)
N1—C2	1.4660 (15)	C5—H5A	0.9600
N1—H1N	0.9200	C5—H5B	0.9600
N2—C1	1.3359 (15)	C5—H5C	0.9600
N2—C4	1.4658 (15)	C6—H6A	0.9600
N2—H2N	0.9199	C6—H6B	0.9600
S1—C1	1.7001 (12)	C6—H6C	0.9600
C2—C3	1.5161 (17)		
			
C4—O1—H1O	104.7	C4—C3—H3B	109.1
C2—O2—H2O	107.2	H3A—C3—H3B	107.9
C1—N1—C2	124.46 (10)	O1—C4—N2	109.54 (10)
C1—N1—H1N	117.0	O1—C4—C6	106.20 (10)
C2—N1—H1N	118.0	N2—C4—C6	109.09 (10)
C1—N2—C4	125.07 (10)	O1—C4—C3	112.36 (10)
C1—N2—H2N	118.2	N2—C4—C3	107.21 (9)
C4—N2—H2N	116.1	C6—C4—C3	112.39 (10)
N2—C1—N1	119.07 (11)	C2—C5—H5A	109.5
N2—C1—S1	119.89 (9)	C2—C5—H5B	109.5
N1—C1—S1	121.04 (9)	H5A—C5—H5B	109.5
O2—C2—N1	109.74 (10)	C2—C5—H5C	109.5
O2—C2—C3	106.53 (10)	H5A—C5—H5C	109.5
N1—C2—C3	107.89 (9)	H5B—C5—H5C	109.5
O2—C2—C5	111.08 (10)	C4—C6—H6A	109.5
N1—C2—C5	108.47 (10)	C4—C6—H6B	109.5
C3—C2—C5	113.05 (11)	H6A—C6—H6B	109.5
C2—C3—C4	112.40 (10)	C4—C6—H6C	109.5
C2—C3—H3A	109.1	H6A—C6—H6C	109.5
C4—C3—H3A	109.1	H6B—C6—H6C	109.5
C2—C3—H3B	109.1		
			
C4—N2—C1—N1	−2.11 (17)	N1—C2—C3—C4	52.12 (13)
C4—N2—C1—S1	178.50 (8)	C5—C2—C3—C4	172.06 (10)
C2—N1—C1—N2	1.77 (18)	C1—N2—C4—O1	−94.88 (13)
C2—N1—C1—S1	−178.85 (9)	C1—N2—C4—C6	149.26 (11)
C1—N1—C2—O2	88.79 (13)	C1—N2—C4—C3	27.30 (15)
C1—N1—C2—C3	−26.90 (16)	C2—C3—C4—O1	68.34 (13)
C1—N1—C2—C5	−149.69 (12)	C2—C3—C4—N2	−52.07 (12)
O2—C2—C3—C4	−65.66 (12)	C2—C3—C4—C6	−171.94 (10)

### Hydrogen-bond geometry (Å, °)

**Table:**

*D*—H···*A*	*D*—H	H···*A*	*D*···*A*	*D*—H···*A*
O1—H1O···O2	0.88	1.98	2.727 (2)	143
O2—H2O···S1^i^	0.88	2.37	3.249 (1)	173
N1—H1N···S1^ii^	0.92	2.60	3.414 (1)	149
N2—H2N···O1^iii^	0.92	2.18	3.074 (2)	164

Symmetry codes: (i) −*x*, −*y*+2, −*z*+1; (ii) −*x*+1, −*y*+2, −*z*+1; (iii) −*x*, −*y*+2, −*z*+2.
